# Negative Regulation of Interferon-β Production by Alternative Splicing of Tumor Necrosis Factor Receptor-Associated Factor 3 in Ducks

**DOI:** 10.3389/fimmu.2018.00409

**Published:** 2018-03-01

**Authors:** Xiaoqin Wei, Wei Qian, Suolang Sizhu, Yongtao Li, Kelei Guo, Meilin Jin, Hongbo Zhou

**Affiliations:** ^1^State Key Laboratory of Agricultural Microbiology, College of Veterinary Medicine, Huazhong Agricultural University, Wuhan, China; ^2^Department of Animal Science, XiZang Agriculture and Animal Husbandry College, Linzhi, China; ^3^College of Animal Husbandry & Veterinary Science, Henan Agricultural University, Zhengzhou, China; ^4^Key Laboratory of Preventive Veterinary Medicine in Hubei Province, The Cooperative Innovation Center for Sustainable Pig Production, Wuhan, China

**Keywords:** duck, duck TRAF3, spliced isoform of duTRAF3, interferon-β, negative regulation, avian virus

## Abstract

Tumor necrosis factor receptor-associated factor 3 (TRAF3), an intracellular signal transducer, is identified as an important component of Toll-like receptors and RIG-I-like receptors induced type I interferon (IFN) signaling pathways. Previous studies have clarified TRAF3 function in mammals, but little is known about the role of TRAF3 in ducks. Here, we cloned and characterized the full-length duck TRAF3 (duTRAF3) gene and an alternatively spliced isoform of duTRAF3 (duTRAF3-S) lacking the fragment encoding amino acids 217–319, from duck embryo fibroblasts (DEFs). We found that duTRAF3 and duTRAF3-S played different roles in regulating IFN-β production in DEFs. duTRAF3 through its TRAF domain interacted with duMAVS or duTRIF, leading to the production of IFN-β. However, duTRAF3-S, containing the TRAF domain, was unable to bind duMAVS or duTRIF due to the intramolecular binding between the *N*- and *C*-terminal of duTRAF3-S that blocked the function of its TRAF domain. Further analysis identified that duTRAF3-S competed with duTRAF3 itself for binding to duTRAF3, perturbing duTRAF3 self-association, which impaired the assembly of duTRAF3-duMAVS/duTRIF complex, ultimately resulted in a reduced production of IFN-β. These findings suggest that duTRAF3 is an important regulator of duck innate immune signaling and reveal a novel mechanism for the negative regulation of IFN-β production *via* changing the formation of the homo-oligomerization of wild molecules, implying a novel regulatory role of truncated proteins.

## Introduction

Tumor necrosis factor receptor-associated factor 3 (TRAF3), a member of the TRAF family, shares a typical structure of TRAFs, a ring finger, five zinc finger motifs, and a TRAF domain (1, 2). The TRAF domain can be divided into an amino-terminal coiled-coil region and a conserved *C*-terminal domain (TRAF-C-domain) (2). The coiled-coil domain of TRAF3 is predicted to be tripartite in structure with three distinct coil regions (3, 4) that are numbered 1 (267–295), 2 (297–339), and 3 (345–367), and the coil 3 is involved in TRAF3 self-association (5).

TRAF3 is a crucial docking protein linking upstream receptor signals to downstream gene activation that regulates varieties of immune signal pathways (6, 7). Recent studies have revealed that TRAF3 negatively regulates MAPK activation and alternative NF-κB signaling, but positively controls type I interferon (IFN) production (8, 9). TRAF3 is essential for Toll-like receptors (TLRs)-dependent and RIG-I-like receptors (RLRs)-dependent type I IFN induction (10, 11). TRAF3 acted downstream of the TLR signaling protein TRIF (for TLR3), whereas TRAF3 acted in the RLRs pathway downstream of adaptor MAVS and then recruited TBK1 and Ikkε to phosphorylate IRF3 to induce IFN-β production. Immunoprecipitation experiments demonstrated that TRAF3 interacts with both MAVS and TRIF (9, 10, 12). Evidence indicates that function of mammalian TRAF3 can be modulated by structurally and functionally distinct isoforms produced by alternative mRNA splicing, and at least eight splice variant isoforms were identified from a TRAF3^+^ lymphoma, including Δ25aa, Δ52aa, Δ56aa, Δ27aa, Δ83aa, Δ103aa, Δ130aa, and Δ221aa (13, 14). In contrast to full-length TRAF3, several shorter TRAF3 splice variants can activate the NF-κB pathway. For example, TRAF3 Δ130, one of splice-deletion variants, induce substantial NF-kB-driven luciferase activity. On the contrary, TRAF3 Δ221 fails to induce NF-kB activation (15). Besides, T cell-specific and activation-dependent alternative splicing generates a Traf3 isoform lacking exon 8 (Traf3DE8) that can activate non-canonical NF-κB signaling. Traf3DE8 disrupts the NIK-TRAF3-TRAF2 complex and allows accumulation of NIK to initiate non-canonical NF-κB signaling and chemokine expression in activated T cells (16). However, the research about the impact of TRAF3 isoforms on IFN-inducing pathways is rare.

In birds, TRAF3 has been found to demonstrate antiviral activity. Chicken TRAF3 is upregulated with Poly(I:C), Poly dA-dT and NDV challenge CEFs, indicating that chicken TRAF3 plays important roles in defending against both RNA and DNA virus infection (17). Pigeon TRAF3 is also induced upon various TLR ligands stimulation (18). TRAF3 is a well-known regulator of the innate antiviral response, but its role in ducks has not been reported. In this study, we identified the full-length duck TRAF3 (duTRAF3) gene and a splice isoform of duTRAF3 (duTRAF3-S) and analyzed the functional role of duTRAF3 and duTRAF3-S in duck immune responses. Overexpression of duTRAF3 inhibited the replication of avian influenza virus (AIV) and duck Tembusu virus (DTMUV), whereas duTRAF3-S increased the replication of AIV and DTMUV. Our data showed that duTRAF3 potentiated IFN-β expression by interacting with duMAVS or duTRIF. However, duTRAF3-S acted as a negative mutant of duTRAF3 and blocked the production of IFN-β *via* disrupting the interactions of duTRAF3-duMAVS/duTRIF. This research brings a better understanding of duck innate immune system.

## Materials and Methods

### Cells, Virus, and Reagents

Duck embryo fibroblasts (DEFs) were prepared from 10-day-old embryonated eggs (purchased from Wuhan Chunjiang Waterfowl Co., Ltd.) and maintained in Dulbecco’s Modified Eagle complete medium (HyClone, China) supplemented with 10% heat-inactivated fetal bovine serum (FBS; Gibco, USA) (19, 20). The human embryonic kidney 293T cell line was purchased from ATCC (Manassas, VA, USA) and was cultured in Roswell Park Memorial Institute-1640 medium (with 10% FBS) (HyClone, China). The cells were transfected with the indicated constructs using Lipofectamine 2000 according to the manufacturer’s instructions (Invitrogen, USA). Low-molecular-weight Poly(I:C) was purchased from Sigma (St. Louis, USA). The recombinant vesicular stomatitis virus encoding green fluorescence protein (VSV-GFP) was a gift from Harbin Veterinary Research Institute. The AIV strain, A/duck/Hubei/hangmei01/2006(H5N1/HM), and a virulent DTMUV strain (GenBank ID: KJ489355) were conserved by the State Key Laboratory of Agricultural Microbiology of China. All experiments with H5N1/HM viruses were performed in Animal Biosafety Level 3 laboratory. This study was carried out in accordance with the recommendations of BSL-3, Huazhong Agricultural University (HZAU). The protocol was approved by the BSL-3 of HZAU. Mouse monoclonal antibodies (mAbs) against Flag-tag, HA tag (Sigma, USA) and GAPDH (Coachella, USA) and rabbit polyclonal antibody against HA tag (PMKbio, China) were used for western blot and indirect immunofluorescence, while Cy3-conjugated goat anti-mouse (Invitrogen) and FITC-conjugated goat anti-rabbit IgG (Invitrogen) were used for indirect immunofluorescence analysis. Rabbit polyclonal antibody against duTRAF3-S was prepared and used for endogenous duTRAF3s expression analysis. HRP-conjugated anti-mouse or anti-rabbit IgG (Jackson Immuno Research Laboratories) were also used for western blot.

### duTRAF3 and duTRAF3-S Cloning, Sequence Alignment, and Homology Analysis

Based on the duck genomic sequence and published predicted sequence of TRAF3 from *Anas platyrhynchos* (GenBank accession number XM_005020702), gene-specific primers (Table S1 in Supplementary Material) for the PCR amplification of the complete coding region were designed using the Primer 5 software. The software package DNAman 6.0 was used to deduce the amino acid sequence. Clustal Omega^1^ and ESPript 3.0^2^ were used to construct multiple sequence alignments of amino acid sequences of TRAF3, and a phylogenetic tree was constructed using the MEGA 6 program. The protein motif analysis was performed using the Sample Modular Architecture Research Tool.^3^

### Animals and Sample Collection

Healthy cherry valley ducks were purchased from Wuhan Chunjiang Waterfowl Co., Ltd. Various tissues of ducks, including the heart, liver, spleen, lung, kidney, brain, pancreas, intestine, trachea, muscle, and stomach, were collected from healthy adult domestic ducks and frozen in liquid nitrogen for further study. Animal experiments were approved by HZAU Animal Care and Use Committee.

### Quantitative Real-time PCR (qRT-PCR)

Total RNA was extracted from DEFs or different tissues of cherry valley ducks by using TRIzol (Invitrogen), following standard instructions. The cDNA was prepared by using AMV reverse transcriptase and an oligo(dT-)18-adaptor primer (TaKaRa, China). cDNAs were used for quantification of the indicated mRNA copy number on an ABI ViiA 7 PCR system (Applied Biosystems, USA) by using SYBR Green Master Mix (Rox). Transcript level of each gene was normalized to the expression of GAPDH, and the 2^−ΔΔCt^ method was used to analyze the gene expression of samples (21). The primers used in qRT-PCR were listed in Table S1 in Supplementary Material.

### Plasmids

Duck TRAF3 (duTRAF3) and duTRAF3-S genes were amplified from duck cDNA (prepared from total RNA extracted from DEFs after poly(I:C) treatment) by PCR and cloned into the EcoRI and XhoI (TaKaRa) sites of the pCAGGS-HA vector with an *N*-terminal HA tag, named HA-duTRAF3 and HA-duTRAF3-S, respectively. pCAGGS-HA expression plasmids encoding truncated duTRAF3 fragments, including HA-T31-216, HA-T321, HA-T3319, HA-Δcoil3, and HA-TRAF-C-domain, were obtained by PCR amplification of HA-duTRAF3. Based on the previous studies (22–25), duRIG-I, duRIG-I(N), duMDA5(N), duMAVS, and duTRIF were amplified from duck cDNA and cloned into the p3xFlag-CMV-14 vector with *C*-terminal Flag epitope tags. Flag-tagged duTLR3 was kindly provided by Dr. Peirong Jiao (Huanan Agricultural University). Plasmids expressing Flag-tagged duTRAF3 and duTRAF3-S were also amplified by PCR and cloned into the p3xFlag vector. Duck IFN-β-luc luciferase reporter plasmid was constructed as previously described (26). Renilla control plasmid pRL-TK was a gift from Dr. Ping Qian (HZAU). For the split firefly luciferase complementation assay, optimal pairs of *N*- and *C*-terminal cDNA fragments (N_Luc_/C_Luc_ combinations of amino acids 2-416/398-550) (27) were amplified by PCR from pGL4.10 plasmid DNA. The *N*- or *C*-terminal cDNA fragment was inserted into the pCAGGS-HA (HA-C_Luc_ and HA-N_Luc_). pCAGGS-HA plasmids encoding N_Luc_-duTRAF3, N_Luc_-duTRAF3-S, duTRAF3-C_Luc_, and duTRAF3-S-C_Luc_ were created by a two-step assembly PCR using plasmid HA-C_Luc_, HA-N_Luc_, HA-duTRAF3, and HA-duTRAF3-S. In an analogous manner, N_Luc_-duTRAF3-C_Luc_ and N_Luc_-duTRAF3-S-C_Luc_ were generated by a two-step assembly PCR using plasmids N_Luc_-duTRAF3, N_Luc_-duTRAF3-S, duTRAF3-C_Luc_, and duTRAF3-S-C_Luc_. All constructs were verified by DNA sequencing (Sangon Biotech Co., Ltd. Shanghai, China). The PCR primers used in this study were summarized in Table S1 in Supplementary Material.

### Luciferase Reporter Assays and Fragment Complementation Assays

Duck embryo fibroblasts seeded into 12-well plates were transfected with IFN-β-luc reporter plasmid and internal control pRL-TK together with other plasmids as indicated. Cells were lysed 24 h later or lysed after cells transfected with Poly(I:C) (400 ng/ml) for 16 h, followed by analysis of cell lysates for luciferase activity with a Dual-Luciferase Reporter Assay System kit (Promega) according to the manufacturer’s protocol. Fragment complementation assays were performed as previously described (27, 28). 293T cells were transfected with plasmids encoding duTRAF3 or duTRAF3-S flanked by the respective fragments of the firefly luciferase or *N*- and *C*-terminal firefly luciferase fused by a linker sequence (29). Cell extract was prepared, and luciferase activity was measured using the Dual-Glo luciferase assay system (Promega).

### RNA Interference

The siRNA oligonucleotides targeting the duTRAF3 unique nucleotide sequence, which was absent in duTRAF3-S, for specific silencing of the full-length duTRAF3, were synthesized by GenePharma. The target sequences are as follows: si-1, 5′-GCGAGUUGAAUGCACAUUU-3′; si-2, 5′-CCAGAAUGAAAGUUUGGAA-3′. The negative control siRNA was obtained from GenePharma. Transfection of siRNA into DEFs was performed by Lipofectamine 2000 according to the manufacturer’s instruction.

### Western Blotting and Co-Immunoprecipitation (Co-IP)

293T cells were transfected with the appropriate plasmids for 24 h. Cells were lysed in lysis buffer [50 mM Tris (pH 8.0), 100 mM NaCl, 20 mM NaF, 50 mM KH_2_PO_4_, 1% Triton X-100, 10% glycerol, and 0.1 mM dithiothreitol] containing the 1 mM phenylmethyl sulfonylfluoride. The lysates were boiled at 100°C for 10 min, then separated by SDS-PAGE, and electroblotted onto a nitrocellulose membrane (BioRad) for western blot assay. The membranes were blocked in 1% bovine serum albumin (BSA) in TBST buffer for 1 h at room temperature and probed with indicated primary Abs for 1 h. After hybridizing with either goat anti-rabbit or goat anti-mouse secondary Abs, the membranes were visualized with ECL reagents (Advansta). For Co-IP analysis, the cells were washed with phosphate-buffered saline (PBS) and lysed for 30 min at 4°C in lysis buffer containing 50 mM TrisHCl (pH 7.4), 150 mM NaCl, 1% NP-40, 10% glycerin, 0.1% SDS, and 2 mM Na_2_EDTA. The cell lysates were then incubated with anti-Flag mAb for 8 h at 4°C, and the complexes were captured using protein A/G plus-agarose (Santa Cruz Biotech). The beads were washed and then analyzed by standard immunoblotting procedures.

### Indirect Immunofluorescence Analysis

Duck embryo fibroblasts were plated onto coverslips in a 12-well plate and transfected with the indicated plasmids (500 ng each). At 24 h posttransfection, cells were washed twice with PBS and fixed in 4% paraformaldehyde for 15 min. Cells were permeabilized with 0.1% Triton X-100 for 15 min and blocked for 1 h at room temperature with 1% BSA in PBS, followed by incubation with the primary antibody for 1 h. After three washes with PBS containing 0.1% Tween 20, cells were incubated with FITC- or Cy3-conjugated secondary antibodies for 1 h and then incubated with 4′,6-diamidino-2-phenylindole (DAPI) (Invitrogen) for 10 min. Finally, coverslips were washed extensively and fixed onto slides. Images were taken under a Zeiss LSM510 Meta confocal microscope (Carl Zeiss, Zena, Germany).

### Virus Infection

Duck embryo fibroblasts were plated in six-well plates. When cells reached approximately 80% confluences, they were transfected with duTRAF3, duTRAF3-S, or empty vector for 24 h, then infected with 0.001 multiplicity of infection (MOI) of H5N1/HM, and infected with DTMUV at an MOI of 0.01. After 1 h of viral adsorption at 37°C, the inoculum was replaced with fresh media, and the cells were incubated at 37°C. Culture supernatants were harvested for measuring viral titers as 50% tissue culture infective dose (TCID_50_).

### Statistical Analysis

The results are expressed as means ± SD. Statistical analyses were performed on data from triplicate experiments by using Student’s *t*-test. *P* < 0.05 was considered significant, and a *P* < 0.01 was considered highly significant.

## Results

### Identification of a Splice Variant of duTRAF3

**Figure 1:**
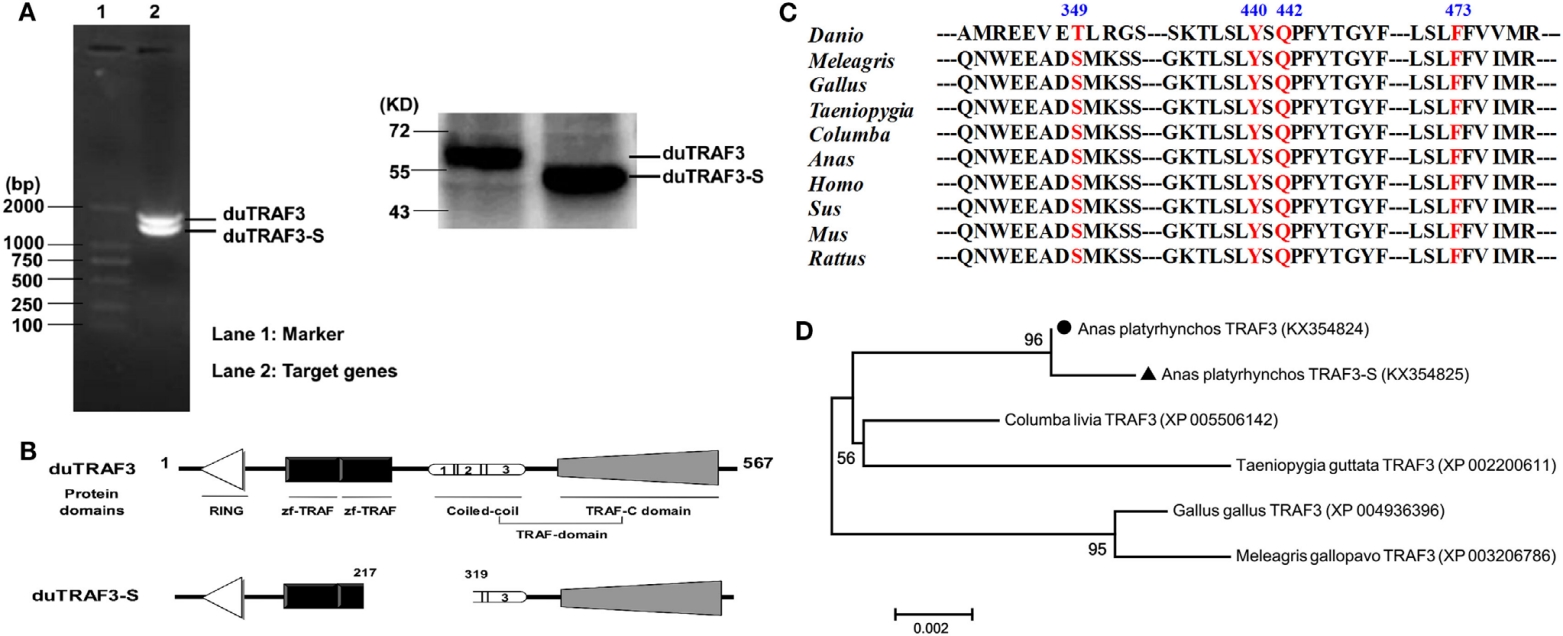
Identification of a splice variant of duck TRAF3 (duTRAF3). **(A)** RT-PCR with duTRAF3-specific primers on RNA isolated from duck embryo fibroblasts and the western blot was used to detect the expression of constructed plasmids in 293T cells. **(B)** Schematic representation of duTRAF3 and spliced isoform of duTRAF3 (duTRAF3-S) protein structure, showing the four different domains. Three distinct coil regions numbered 1 (267-295), 2 (297-339), and 3 (345-367). **(C)** A cross-species sequence alignments of TRAF3 revealed the high conservation of important fragments. **(D)** Phylogenetic analysis of avian TRAF3. The tree was constructed by the neighbor-joining method using amino acid sequences aligned with MEGA 6. The scale bar indicated a branch length of 0.002.

The full-length duTRAF3 PCR product was obtained from duck cDNA by using specific primers (Table S1 in Supplementary Material) that were designed on the predicted coding sequence of duTRAF3 (GenBank accession number XM_005020702). During cloning of the full-length duTRAF3, a short sequence, duTRAF3-S was identified (Figure 1A). duTRAF3 gene contained 1701bp and encoded 567 aa residues. The deduced protein structure prediction of duTRAF3 contained a ring finger, two TRAF-type zinc finger motifs, and a TRAF domain, which contained a coiled-coil domain and a conserved TRAF-C domain (Figure 1B). The coiled-coil domain of duTRAF3 was numbered 1 (267–295), 2 (297–339), and 3 (345–367) (5). This confirmed that duTRAF3 had a high degree of structural similarity to mammalian TRAF3. duTRAF3-S lacked a portion of zinc finger domain, a complete of coil 1 domain, and a portion of coil 2 domain from position 217–319 (Figure 1B; Figure S1 in Supplementary Material). The obtained duTRAF3 and duTRAF3-S sequences were deposited in GenBank (accession number KX354824 and KX354825, respectively). Alignment of the full-length sequence indicated that duTRAF3 shared a high level of identity with other species (88–99%) (Figure S2 in Supplementary Material), and several residues showed to be important for the function of human TRAF3 were conserved in ducks, including Ser349 that was a phosphorylation site in TRAF3 (30), Tyr440, and Phe473 (Figure 1C), which were contributed to MAVS recognition (31). A phylogenetic tree was constructed based on the multiple alignments of duTRAF3, duTRAF3-S, and TRAF3s from other species (Figure S3 in Supplementary Material). The sequence alignment results displayed that TRAF3s in birds clustered in one group, and duTRAF3 and duTRAF3-S were clustered together and then converged into a clade with Columba livia TRAF3 (Figure 1D).

### duTRAF3s mRNAs Tissue-Specific Expression

**Figure 2:**
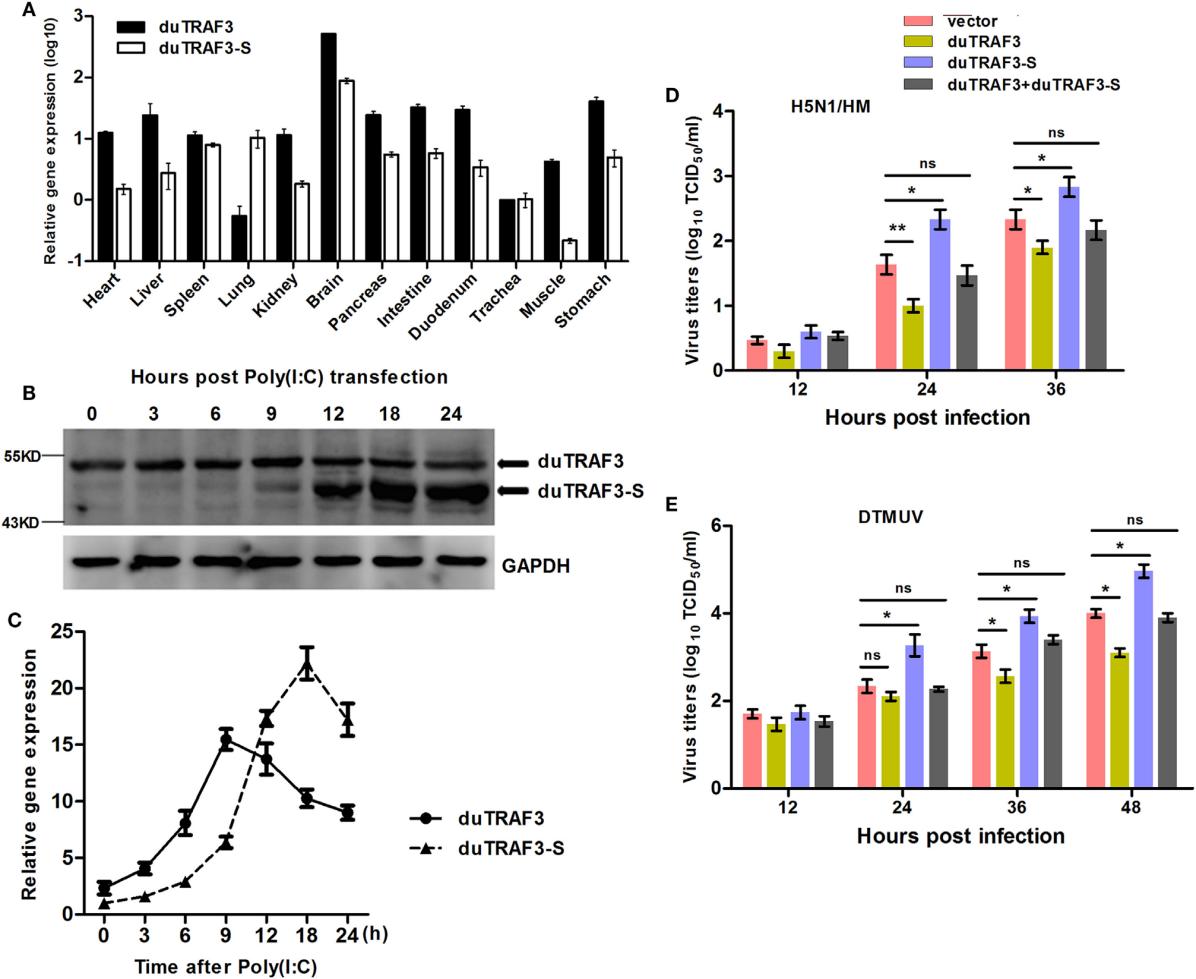
Analysis of expression patterns of duck TRAF3s (duTRAF3s) and the effects on viral replication. **(A)** Constitutive expression of duTRAF3 and spliced isoform of duTRAF3 (duTRAF3-S) transcripts in healthy duck tissues were performed by quantitative real-time PCR (qRT-PCR). The mRNA expression levels were expressed as relative mRNA indexes, calculated as index (duTRAF3 mRNA or duTRAF3-S mRNA copy number/GAPDH mRNA copy number) of test tissue divided by index of trachea. **(B,C)** Duck embryo fibroblasts (DEFs) were treated with poly(I:C) (2 µg/ml) for the indicated time points. The mRNA and protein levels of duTRAF3 were detected. **(D,E)** DEFs were transfected with duTRAF3, duTRAF3-S, duTRAF3, and duTRAF3-S, or an empty vector for 24 h and then infected with H5N1/HM virus at multiplicity of infection (MOI) of 0.001 or duck Tembusu virus (DTMUV) at MOI of 1. Culture supernatants were collected for detecting the viral titers, which was measured by TCID_50_ assay. All data were presented as the means ± SD derived from three repeat experiments. *P < 0.05 or **P < 0.01 (Student’s *t*-test).

The abundance of duTRAF3s transcripts in normal tissues was analyzed by qRT-PCR. The qduTRAF3/qduTRAF3-S-F/R primers (Table S1 in Supplementary Material) were designed to distinguish duTRAF3 and duTRAF3-S. The mRNAs of duTRAF3 and duTRAF3-S were detected in all examined tissues. Both duTRAF3 and duTRAF3-S were expressed a highest level in brain, and the expression of duTRAF3 was higher than duTRAF3-S in all tissues except lungs. duTRAF3 was expressed at high level in the liver and digestive system (intestine pancreas, duodenum, and stomach) (Figure 2A).

### Upregulation of duTRAF3s Expression under Virus Analog Challenge

To detect the expression of endogenous duTRAF3s under stimulation, we generated a rabbit polyclonal Ab to duTRAF3-S protein. Due to duTRAF3-S, which has no unique nucleotide sequence compared with duTRAF3, western blotting showed that the anti-duTRAF3-S Ab recognized overexpressed duTRAF3 protein and duTRAF3-S protein (data not shown). To determine the expression patterns of endogenous duTRAF3 and duTRAF3-S, DEFs were transfected with poly(I:C), then the protein levels of duTRAF3 and duTRAF3-S were detected at various time points by western blot using anti-duTRAF3-S Ab. As shown in Figure 2B, considerable duTRAF3 protein was detected following poly(I:C) stimulation, with peak levels at 9 h posttransfection and a subsequent decrease in duTRAF3 protein levels being observed by 12 h posttransfection. However, the levels of duTRAF3-S protein can be neglected until 12 h posttransfection and then persisted at high levels. This phenomenon is also observed at the transcription level of duTRAFs (Figure 2C). These data indicated that during poly(I:C) stimulation, the expression levels of duTRAF3 and duTRAF3-S were regulated in DEFs.

### The Effects of Overexpressed duTRAF3s on the Replication of Different Viruses

We first examined the impact of duTRAF3s overexpression on avian virus. When infected with AVI H5N1/HM, the progeny virus titers in supernatants of duTRAF3-overexpressing DEFs were significantly decreased compared with control cells, while duTRAF3-S overexpression increased viral replication (Figure 2D). In addition, for another avian virus DTMUV infection, cells expressing duTRAF3 were substantially less susceptible than cells that express a control vector, whereas significantly higher virus titers were observed in duTRAF3-S-expressing cells (Figure 2E). However, replication of H5N1/HM and DTMUV viruses replication were no significant difference when coexpression of duTRAF3 and duTRAF3-S compared with control group (Figures 2D,E). Altogether, these data demonstrate that ectopic expression of duTRAF3s affect avian virus propagation. duTRAF3 induced an antiviral state in DEFs cells, while duTRAF3-S promoted virus replication in DEFs.

### duTRAF3 Induces Expression of IFN-β, While duTRAF3-S Inhibits IFN-β Production

**Figure 3:**
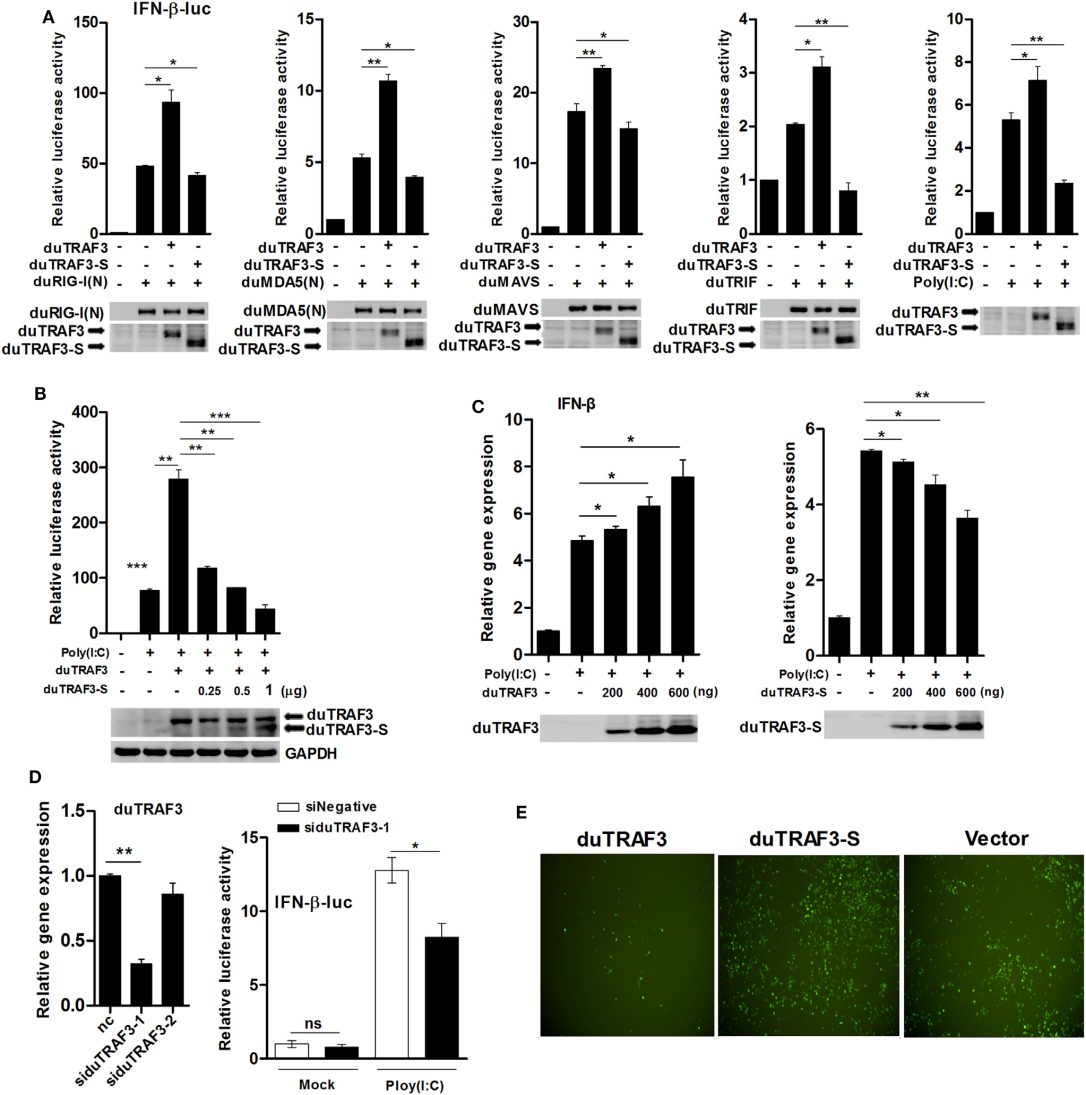
Duck TRAF3s (duTRAF3s) activated interferon (IFN)-β transcription, and spliced isoform of duTRAF3 (duTRAF3-S) inhibited IFN-β activation. **(A)** Duck embryo fibroblasts (DEFs) were transfected with an empty vector, duTRAF3, or duTRAF3-S along with IFN-β-luc and pRL-TK for 24 h, and cells were then transfected with duRIG-I(N), duMDA5(N), duMAVS, or duTRIF for 24 h or transfected with poly(I:C) for 16 h. **(B)** DEFs were transfected with duTRAF3 or an increasing amount of duTRAF3-S as indicated for 24 h and then transfected with poly(I:C) for 16 h. Then the luciferase activity was detected. **(C)** DEFs were transfected with increasing amounts of plasmids encoding duTRAF3 and duTRAF3-S (200, 400, 600 ng) for 24 h, cells were transfected with poly(I:C) for 16 h, and then the transcription of IFN-β was measured. Western blot analysis of lysates was performed. **(D)** DEFs were transfected with two duTRAF3 siRNA (60 pmol) for 24 h, and then total RNAs were extracted to examine the mRNA levels of duTRAF3 transcripts by qPCR. DEFs were transfected with negative control siRNA or duTRAF3 siRNA (si-1) together with IFN-β reporter and pRL-TK plasmids for 24 h, then transfected with poly(I:C), and luciferase activity was measured at 16 h posttransfection. The relative firefly luciferase activity was normalized to the renilla luciferase activity, and the untreated empty vector control value was set to 1. Data are represented as mean ± SD. Experiments were repeated for three times. Student’s *t*-test was performed to determine the *P* value. *P < 0.05; **P < 0.01; ***P < 0.001. **(E)** DEFs were transfected with expression plasmids for duTRAF3, duTRAF3-S, or empty vector. At 24 h after transfection, the cells were infected with vesicular stomatitis virus encoding green fluorescence protein (VSV-GFP) [multiplicity of infection (MOI) of 0.1]. Twelve hours later, GFP expression was visualized by fluorescent microscopy.

Previously, it has been shown that TRAF3 in mammals was a scaffold protein that interacted with components of cellular TLRs and RLRs signaling pathways to induce the production of type I IFN (32). To investigate whether duTRAF3 and duTRAF3-S are involved in duck type I IFN production pathway, DEFs were cotransfected with duTRAF3 or duTRAF3-S and a series of expression constructs encoding the molecules of RLRs signaling pathway or TLRs signaling pathway, including duRIG-I(N), duMDA5(N), duMAVS and duTRIF, or poly(I:C) (TLR3 agonist). Transfection of DEFs with duRIG-I(N), duMDA5(N), duTRIF, duMAVS, or poly(I:C) activated the IFN-β promoter activity, consistent with previous studies (22, 33–35). Reporter assays showed that coexpression of duTRAF3 reproducibly enhanced the level of IFN-β promoter activity to constitutively activated cells expressing plasmids (Figure 3A) as has been demonstrated in human TRAF3 (10). In contrast, duTRAF3-S overexpression inhibited duRIG-I(N)-, duMDA5(N)-, duTRIF-, duMAVS-, and poly(I:C)-mediated IFN-β promoter activation (Figure 3A). Moreover, duTRAF3-S dose dependently inhibited the ability of duTRAF3 to enhance the poly(I:C)-induced IFN-β promoter activity (Figure 3B). We next examined the ability of the duTRAF3 and duTRAF3-S to influence the expression of IFN-β mRNA. As expected, duTRAF3 overexpression increased poly(I:C)-induced production of IFN-β mRNA in a dose-dependent manner. In contrast, overexpression of duTRAF3-S resulted in inhibiting poly(I:C)-induced production of IFN-β mRNA also in a dose-dependent manner (Figure 3C). The role of endogenous duTRAF3 in IFN-β induction to be further confirmed by knocking it down using specific siRNAs targeting aa 217-319, which was absent in duTRAF3-S. The results showed that siduTRAF3-1 knockdown was the most effective, leading to a 68% decrease in duTRAF3 mRNA level (Figure 3D). Therefore, siduTRAF3-1 was selected for subsequent experiments. Luciferase reporter assays showed that knockdown of duTRAF3 expression reduced poly(I:C)-induced IFN-β promoter activity compared to that in the si-negative group (Figure 3D).

Vesicular stomatitis virus is very sensitive to IFN-β, when the host cells were pretested with IFN-β, and VSV growth was inhibited (36–38). To further analyze the contribution of duTRAF3s in IFN-β induction, DEFs overexpressed the duTRAF3 or duTRAF3-S and then were infected with VSV-GFP. DEFs overexpressing duTRAF3 were significantly resistant to VSV infection. However, the GFP fluorescence was brighter after duTRAF3-S overexpression (Figure 3E). These results confirmed that duTRAF3 induced IFN-β production, but duTRAF3-S inhibited the production of IFN-β.

### duMAVS or duTRIF Associates with duTRAF3, but not duTRAF3-S

**Figure 4:**
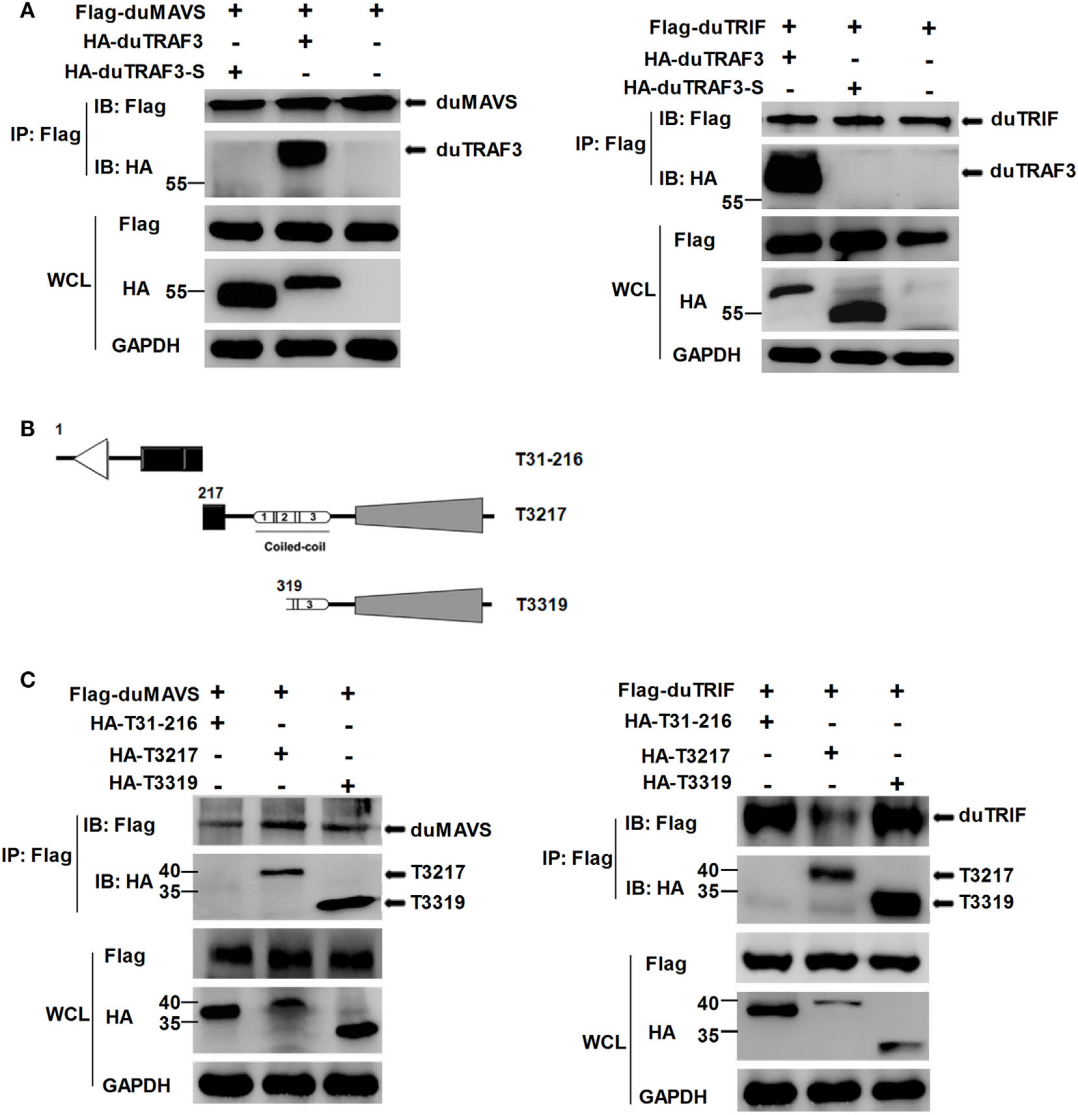
duMAVS or duTRIF interact with duck TRAF3 (duTRAF3) and the TRAF domain of duTRAF3, but not spliced isoform of duTRAF3 (duTRAF3-S). **(A)** Plasmids encoding Flag-tagged duMAVS or duTRIF were cotransfected with HA-tagged duTRAF3 or duTRAF3-S in 293T cells. The cells were lysed 24 h after transfection and subjected to immunoprecipitation with anti-Flag antibody. The immunoprecipitation complexes were analyzed by western blotting using anti-Flag and anti-HA antibodies. **(B)** Schematic map of the duTRAF3 mutants (T31-216, T3217, T3319). **(C)** Cells were cotransfected Flag-tagged duMAVS or duTRIF with HA-tagged different duTRAF3 mutants, and then immunoblotting analysis was performed as described above. Co-Immunoprecipitation (Co-IP) was performed with anti-FLAG, and the precipitates were probed with anti-Flag and anti-HA.

To explore the mechanism by which duTRAF3-S inhibited IFN-β production, we first determined whether the inhibition of IFN-β production by dUTARF3-S was due to the functional defect of duTRAF3-S. As shown in Figure 4A, duTRAF3 was coprecipitated with duMAVS or duTRIF, suggesting that duTRAF3 had an interaction with duMAVS or duTRIF, consistent with human TRAF3. But duTRAF3-S was unable to interact with duMAVS or duTRIF. Previous studies have demonstrated that the TRAF domain of human TRAF3 was capable of binding to signal molecules (12). Three truncations of duTRAF3 lacking the different domains were constructed for Co-IP experiments (Figure 4B). T3217 and T3319 truncations, containing the TRAF domain, were coprecipitated with duMAVS or duTRIF. These results indicated that duMAVS and duTRIF had an association with the TRAF domain of duTRAF3 (Figure 4C), consistent with previous findings in human TRAF3.

### Conformation Change of duTRAF3-S

**Figure 5:**
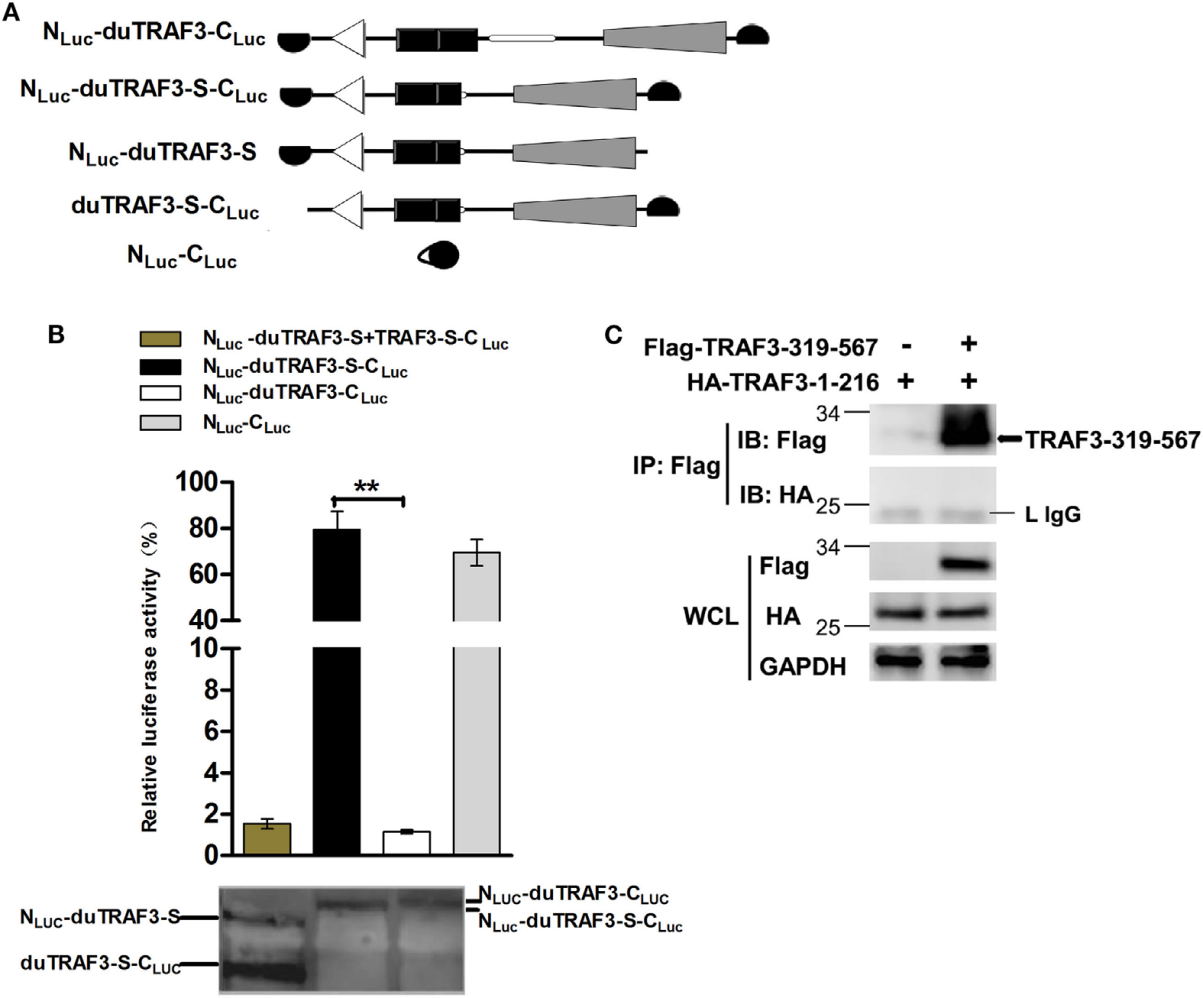
Spliced isoform of duTRAF3 (duTRAF3-S) has an intramolecular interaction between its *N*- and *C*-terminal domains. **(A)** Diagram depicting duck TRAF3 (duTRAF3) or duTRAF3-S fused to split firefly luciferase halves highlighted in black. **(B)** To analyze duTRAF3-S intramolecular folding events, 293T cells were transfected with plasmids encoding N_Luc_-duTRAF3-C_Luc_, N_Luc_-duTRAF3-S-C_Luc_, or plasmids expressing both N_Luc_-duTRAF3-S and duTRAF3-S-C_Luc_ or N_Luc_-C_Luc_ as a control. Western blot analysis of lysates was performed. Error bars indicate SDs of three independent experiments. Student’s *t*-test was performed to determine the *P* value. **P < 0.01. **(C)** 293T cells were cotransfected with HA-duTRAF3-1-216 and Flag-TRAF3-319-567 for 24 h. Cell lysates were harvested, then subjected to immunoprecipitation using an anti-Flag antibody, and analyzed by western blotting.

Spliced isoform of duTRAF3 (duTRAF3-S) failed to interact with duMAVS or duTRIF even though duTRAF3-S had the TRAF domain. Therefore, we speculated that a conformation change of duTRAF3-S might block the function of its TRAF domain. To confirm the conjecture, both ends of duTRAF3-S or duTRAF3 were fused to split firefly luciferase halves (N_Luc_-duTRAF3-C_Luc_, N_Luc_-duTRAF3-S-C_Luc_) to perform fragment complementation assays (Figure 5A). The backfolding of the *N* terminus to the *C* terminus would bring the split firefly luciferase moieties into close proximity and allow reversible reconstitution of active luciferase. In contrast, a conformation that prevents the close proximity of split firefly luciferase moieties would result in no reporter activity (39). The luciferase activity observed after the expression of N_Luc_-duTRAF3-S-C_Luc_ was significantly higher (>80-fold) than with N_Luc_-duTRAF3-C_Luc_ and was equal to the positive group (N_Luc_-C_Luc_) (Figure 5B). To confirm that the signals we measured in the split-luciferase assay did result from an intramolecular rather than intermolecular association between two duTRAF3-Ss, we expressed both duTRAF3-S fused to the *N*-terminal domain of split firefly luciferase (N_Luc_-duTRAF3-S) and duTRAF3-S fused to the *C*-terminal domain of firefly luciferase (duTRAF3-S-C_Luc_) and compared the luciferase activity obtained with cell extract containing N_Luc_-duTRAF3-S-C_Luc_ only. As expected, the luciferase activity observed after expression of both N_Luc_-duTRAF3-S and duTRAF3-S-C_Luc_ was significantly lower (>80-fold) than with N_Luc_-duTRAF3-S-C_Luc_ (Figure 5B), indicating that the activity with the duTRAF3-S was indeed an intramolecular closing between the *N*- and *C*-terminal domains of duTRAF3-S.

To test the binding between the *N*- and *C*-terminal domains of duTRAF3-S, we examined the association of duTRAF3-1-216 and TRAF3-319-567. Unfortunately, the interaction between this two truncations was not detected (Figure 5C). Thus, the conformation change of duTRAF3-S bring *N*-terminal domain into close proximity to *C*-terminal domain, which impairs the functional role of duTRAF3-S in innate immune signaling pathway.

### duTRAF3 Self-Association Is Required for the Binding of duTRAF3 to duMAVS or duTRIF

**Figure 6:**
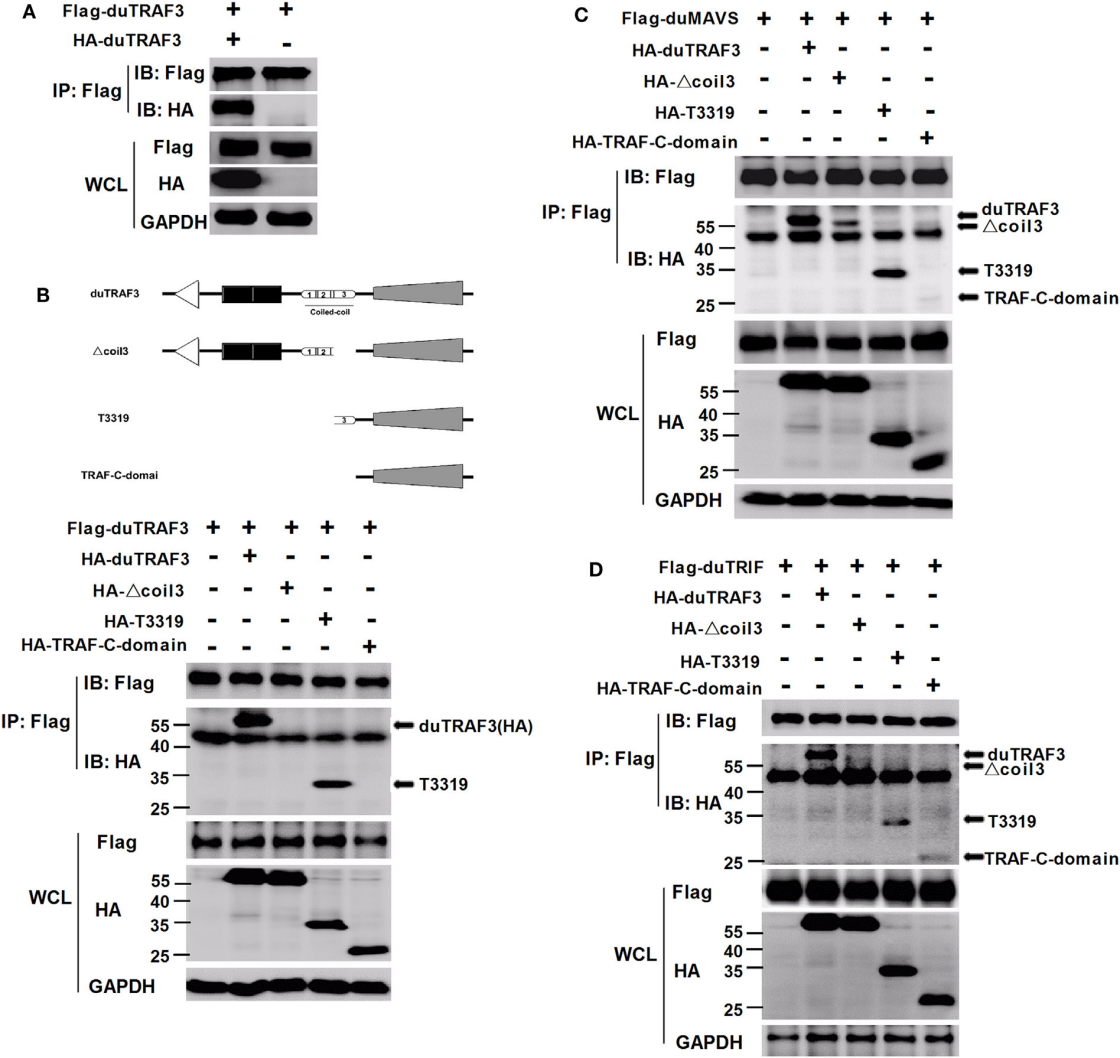
Duck TRAF3 (duTRAF3) self-association requires its coil 3 region and is required for the binding of duTRAF3 to duMAVS or duTRIF. **(A)** 293T cells were cotransfected with Flag-duTRAF3 and HA-duTRAF3. **(B)** Schematic map of the duTRAF3 and the deletion mutants [duTRAF3, Δcoil 3, T3319, and conserved *C*-terminal domain of TRAF (TRAF-C-domain)]. 293 T cells were transfected with Flag-duTRAF3 together with HA-duTRAF3, HA-Δcoil 3, HA-T3319, or HA-TRAF-C-domain. **(C,D)** 293T were cotransfected with Flag-duMAVS **(C)** or Flag-duTRIF **(D)** together with HA-tagged duTRAF3 or duTRAF3 truncation forms. Twenty-four hours after transfection, cells were collected and subjected to immunoprecipitation with anti-Flag. The immunoprecipitation complexes were analyzed by western blotting using anti-Flag and anti-HA antibodies.

Duck TRAF3 (duTRAF3) formed homo-oligomerization (Figure 6A), consistent with previous findings in human TRAF3 (5, 40). Force et al. identified self-association of human TRAF3 required its coil 3 region (5). To investigate how deletion of coil 3 region of duTRAF3 influenced the duTRAF3 self-oligomerization, Flag-duTRAF3 was overexpressed in 293T cells along with several different duTRAF3 deletion mutants. duTRAF3 coprecipitated with duTRAF3 and T3319; however, mutants lacking coil 3, Δcoil 3 that deleted coil3 region from the full-length duTRAF3 and TRAF-C-domain that deleted coil3 region from the TRAF domain (T3319), lost the ability to oligomerize with duTRAF3 (Figure 6B). Thus, coil 3 region was required for duTRAF3 self-association. A recent crystal structure analysis revealed that MAVS recruited TRAF3 homotrimers to activate the induction of IFN (31). To determine whether duTRAF3 homo-oligomers formation influenced the interaction between duTRAF3 and duMAVS or duTRIF, duMAVS, or duTRIF was overexpressed in 293T cells along with duTRAF3 deletion mutants. Δcoil 3 and TRAF-C-domain that lost the ability to form homo-oligomers bound only very low amounts of duMAVS/duTRIF (Figures 6C,D), so the coil 3 region was important for the binding of TRAF domain to duMAVS/duTRIF. Consequently, we concluded that duTRAF3 homo-oligomerization was critical for the binding of duTRAF3 to duMAVS/duTRIF.

### duTRAF3-S Blocks the Interaction of duTRAF3 to duMAVS or duTRIF

**Figure 7:**
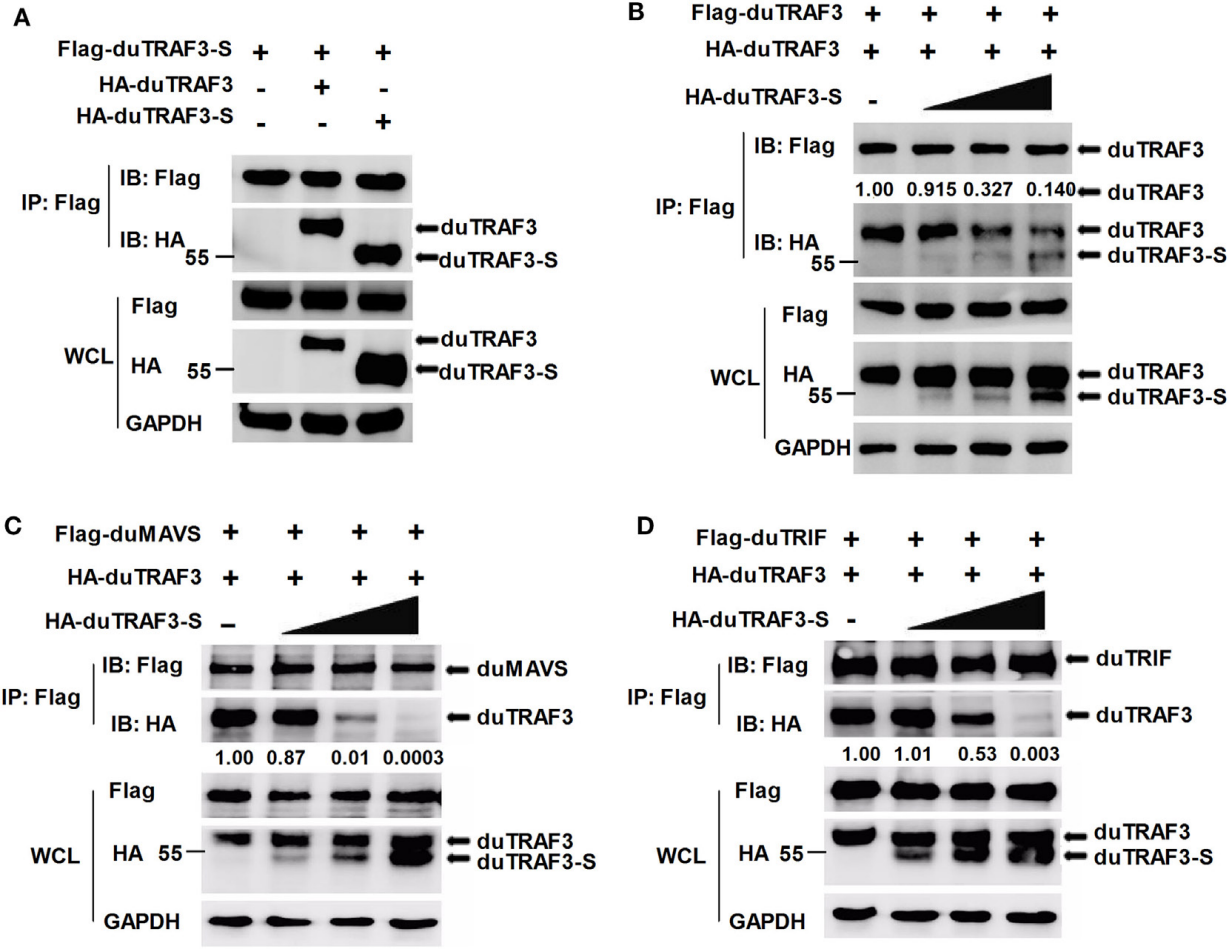
Spliced isoform of duck TRAF3 (duTRAF3-S) forms a hetero-oligomerization with duck TRAF3 (duTRAF3) and disrupts the interaction between duTRAF3 and duMAVS or duTRIF. **(A)** 293T cells were transfected with Flag-tagged duTRAF3-S and HA-tagged duTRAF3 or duTRAF3-S. **(B–D)** 293T cells were transiently transfected with Flag-duTRAF3 **(B)** or Flag-duMAVS **(C)** or Flag-duTRIF **(D)** and HA-TRAF3 together with an increasing amount of HA-duTRAF3-S (0.5, 1, 2 µg). Twenty-four hours after transfection, the cell lysates were collected and subjected to immunoprecipitation and immunoblotting using the indicated antibodies. Co-Immunoprecipitation (Co-IP) was performed with anti-Flag, and the precipitates were probed with anti-Flag and anti-HA. The protein values were calculated by using Image-Pro Plus software.

As duTRAF3-S retains the coil 3 region, we sought to determine whether duTRAF3-S could interact with duTRAF3. Figure 7A shows that duTRAF3-S formed homo-oligomerization and interacted with duTRAF3, which formed hetero-oligomerization. Increasing the expression of duTRAF3-S reduced duTARF3 self-association and then inhibited duTRAF3 homo-oligomers formation (Figure 7B). These results indicated that duTRAF3-S was capable of forming a hetero-oligomerization with duTRAF3 for inhibiting duTRAF3 self-association.

Given that duTRAF3-S was incapable of binding to duMAVS and duTRIF and duTRAF3 self-association was required for the binding of duTRAF3 to duMAVS/duTRIF, a hetero-oligomerization between duTRAF3-S and duTRAF3, which blocked the duTRAF3 self-association, is likely to impair the interaction between duTRAF3 and duMAVS/duTRIF. To further confirm our speculation, we performed an additional experiment, 293T cells were cotransfected with duTRAF3 and duTRAF3-S, and the binding of duTRAF3 to duMAVS or duTRIF was examined by Co-IP. With the increase of the expression level of duTRAF3-S, the formation of duMAVS/duTRIF-TRAF3 complex was severely impaired (Figures 7C,D). These data indicated that duTRAF3-S disrupted the interaction between duMAVS/duTRIF and duTRAF3 by forming a hetero-oligomerization with duTRAF3.

### Localization of duTARF3s

**Figure 8:**
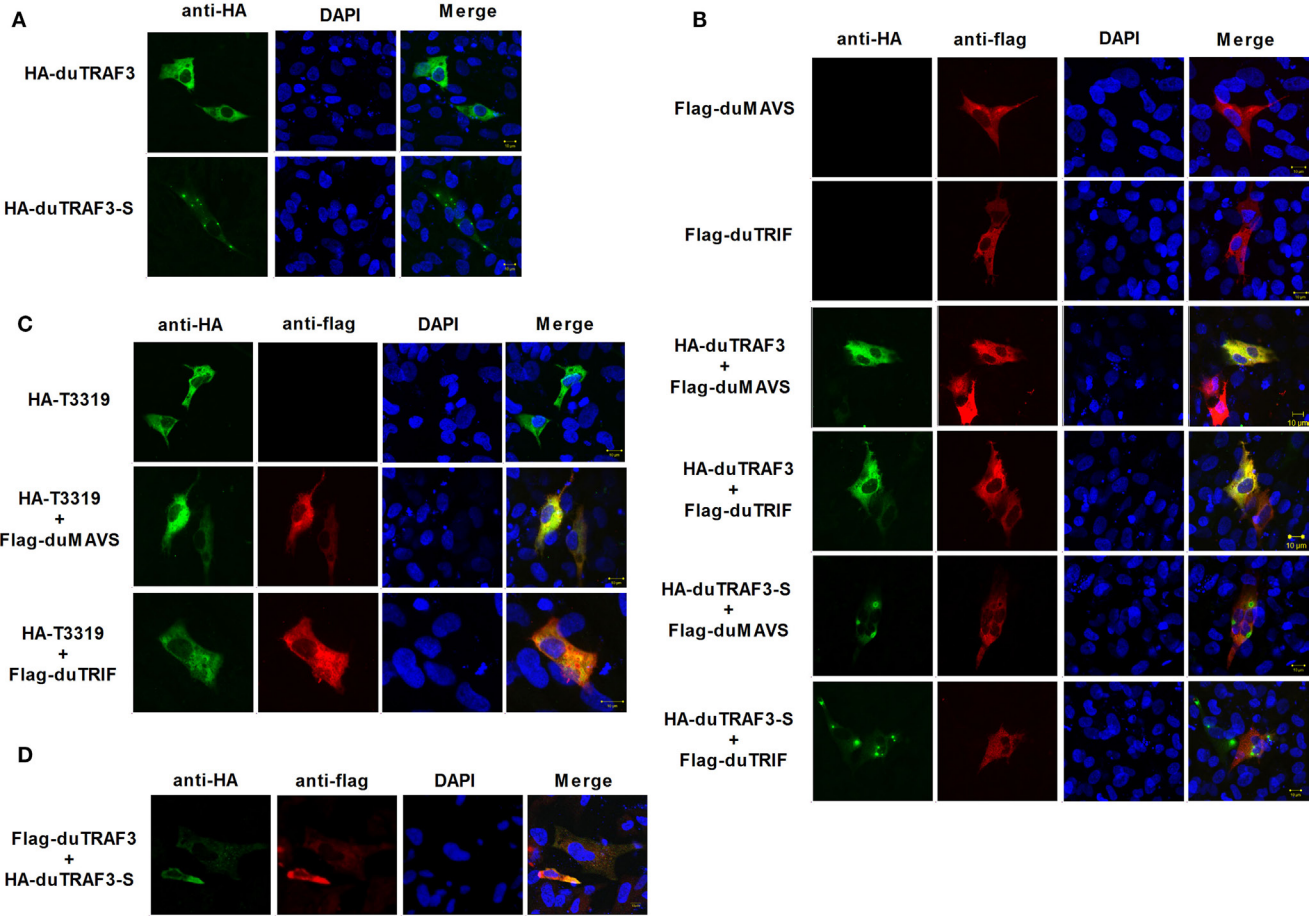
duMAVS or duTRIF colocalizes with duck TRAF3 (duTRAF3) or TRAF domain of duTRAF3 in the cytoplasm, but not spliced isoform of duTRAF3 (duTRAf3-S). **(A)** Duck embryo fibroblasts (DEFs) seeded in 12-well plates were transiently transfected individually with expression plasmids for HA-duTRAF3 or HA-duTRAF3-S for 24 h, and cells were then fixed and stained with antibody against the HA tag. Subsequently, cells were treated with FITC-conjugated goat anti-mouse secondary antibody and with 4′,6-diamidino-2-phenylindole (DAPI). **(B)** DEFs were transfected with Flag-duMAVS or Flag-duTRIF and HA-duTRAF3 or HA-duTRAF3-S. **(C)** DEFs transiently transfected with HA-T3319 and Flag-duMAVS or Flag-duTRIF. **(D)** DEFs transiently transfected with HA-duTRAF3-S and Flag-duTRAF3. Twenty-four hours after transfection, cells were fixed with 4% paraformaldehyde and stained with anti-HA or anti-Flag antibody. Secondary antibodies conjugated to Cy3 or FITC were used to visualize the stained Flag-tagged and HA-tagged proteins, respectively. DAPI show the nuclei of cells. Fluorescence was examined with a Zeiss LSM 510 Meta confocal microscope.

Duck TRAF3 (duTRAF3) and duTRAF3-S had different cellular localization patterns, and duTRAF3 was uniformly distributed throughout the cytoplasm, whereas duTRAF3-S protein was expressed throughout the cytoplasm with some granular structures (Figure 8A). duTRAF3 and duMAVS or duTRIF were found to be colocalized in the cytoplasm. In contrast, duTRAF3-S failed to colocalize with duMAVS or duTRIF (Figure 8B). The TRAF domain of duTRAF3 was also found to be colocalized with duMAVS or duTRIF (Figure 8C). These results were consistent with Co-IP assays. Furthermore, duTRAF3 and duTRAF3-S were found to be colocalized in the cytoplasm, and duTRAF3 overexpression led to a uniform distribution of duTRAF3-S (Figure 8D).

## Discussion

In previous studies, alternative splicing is widely recognized as a mechanism to increase the coding capacity of the genome and to have a large regulatory potential (41). Several isoforms of TRAF3 have been described, and only partial functions have been studied in mammals (15, 16, 42). In contrast to full-length TRAF3, several shorter TRAF3 splice variants disrupt the NIK-TRAF3-TRAF2 complex and allow accumulation of NIK to initiate non-canonical NF-κB signaling. However, little is known about the information of duTRAF3 or its isoforms in ducks. In this study, we cloned duTRAF3 gene and duTRAF3-S gene that an alternative isoform with 103 aa deletion of duTRAF3-S. Protein structure, multiple alignments, and phylogenetic analysis (Figure 1) indicated that duTRAF3 was a true orthologous of mammalian TRAF3 involved in regulation of IFN expression in mammalians, and we identified that the endogenous duTRAF3 and duTRAF3-S were expressed in DEFs (Figure 2B). In this study, we have shown that duTRAF3-S inhibited the production of IFN-β by disrupting the formation of duMAVS/duTRIF-duTRAF3 complex.

**Figure 9:**
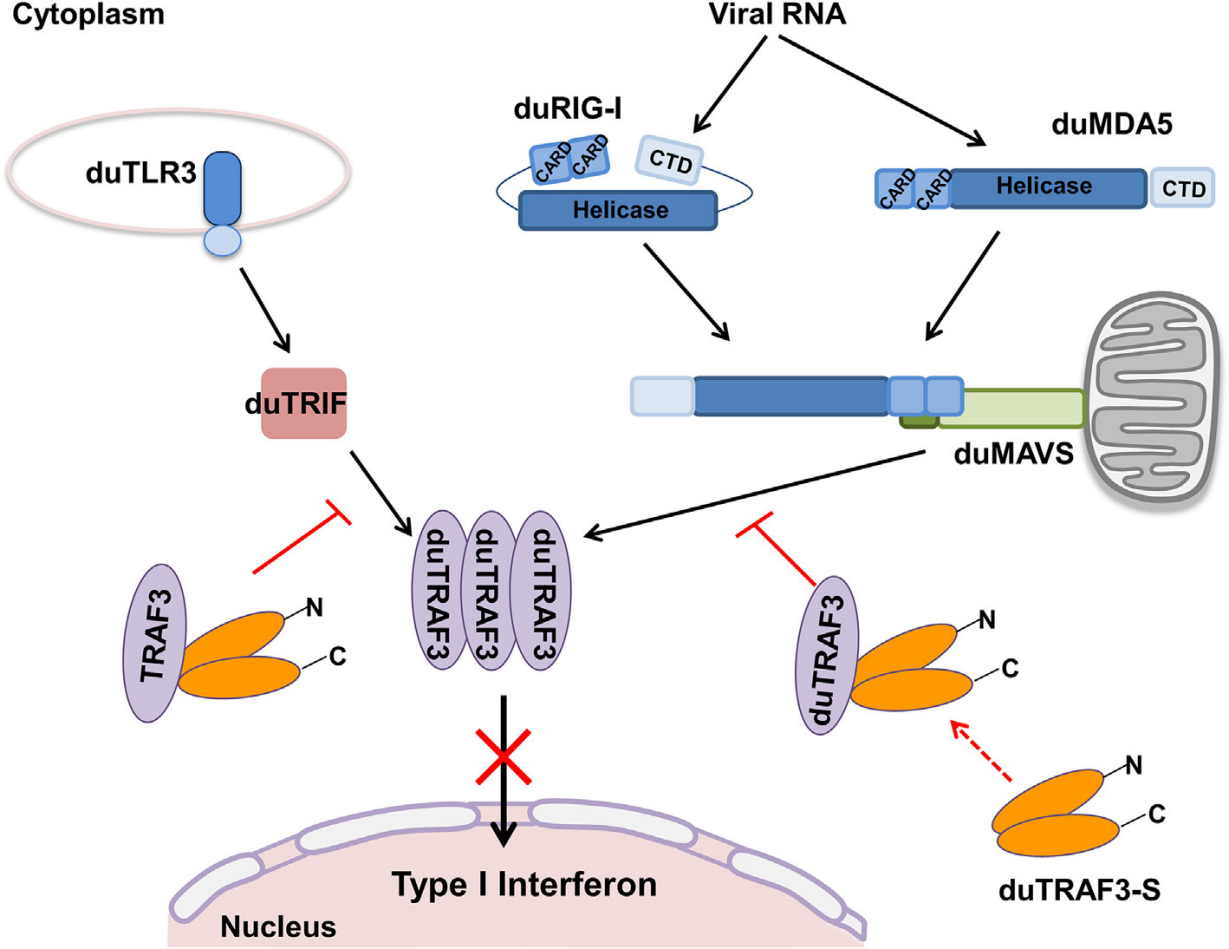
Model for regulation of type I interferon signaling pathway by duck TRAF3s (duTRAF3s) in duck cells. In duck RIG-I-like receptors, signaling pathway, after duRIG-I/duMDA5 recognizes pathogen-associated molecular patterns (PAMPs), results in the recruitment of duMAVS, which in turn recruits duTRAF3 homotrimers and then induces the activation of type I interferon response; following recognition of PAMPs, duTLR3 interacts with duTRIF, and then duTRIF recruits homotrimeric duTRAF3 proteins to activate type I interferon promoter. Spliced isoform of duTRAF3 (duTRAF3-S) interacts with duTRAF3, which impairs the homotrimeric structural of duTRAF3. The formation of duTRAF3–duTRAF3-S hetero-oligomerization blocks the interaction between duTRAF3 and duMAVS or duTRIF and then attenuates the activation of type I interferon pathway.

Toll-like receptors and RLRs are two main PRRs in the type I IFN signaling pathway (43). Although TLRs and RLRs recruit different downstream adaptors for IFN signaling, both use TRAF3 to transmit signals to downstream signaling molecules; therefore, TRAF3 plays an important role in the regulation of IFN-β response events. We found that knockdown of duTRAF3 significantly blocked the IFN-β production induced by poly(I:C) (Figure 3). Unfortunately, because duTRAF3-S has no unique nucleotide sequence compared with duTRAF3, we could not design specific siRNA to target duTRAF3-S. Luciferase reporter gene assays demonstrated that duTRAF3 enhanced IFN-β production, while duTRAF3-S inhibited IFN-β production. Further study showed that duTRAF3 through its TRAF domain associated with duMAVS/duTRIF (Figure 4C), which were consistent with mammalian TRAF3. The intramolecular folding between the N- and C-terminal domains of duTRAF3-S (Figure 5B), which might cause the TRAF domain to be wrapped by *N*-terminal of duTRAF3-S, leading to duTRAF3-S fails to interact with duMAVS/duTRIF (Figure 4A). So, the 103aa deletion brought a real structural difference between duTRAF3-S and duTRAF3. Therefore, it was not surprising that duTRAF3-S had functional defect. A series of crystal structures of TRAF3 complexes revealed that TRAF3 formed a homotrimer with the typical mushroom-like structure and function as a trimer in solution (2, 31, 44–48). The trimer was stabilized mainly by coiled–coil interaction (45). Evidence suggested that R118W mutant of TRAF3 destabilized the TRAF3 trimer formation, which resulted in the functional defect in TRAF3 (49, 50). A recent crystal structure analysis revealed that MAVS homo-oligomers recruited TRAF3 homotrimers to activate the induction of IFN (31, 51). According to these researches, it could be deduced that the activation of TRAF3 required its oligomerization. Previous study determined that coil 3 region was critical for the self-association of TRAF3 into oligomers (5). Our research proved a self-association of duTRAF3 (Figure 6A) and coil 3 was required for duTRAF3 self-association to form homo-oligomerization (Figure 6B). The duTRAF3 homo-oligomerization was required to efficiently form duMAVS/duTRIF-duTRAF3 complex (Figures 6C,D). duTRAF3-S contained the coil 3 region, which indicated duTRAF3-S can form oligomers. We confirmed duTRAF3-S associate with duTRAF3 to form hetero-oligomerization (Figure 7A). We found duTRAF3-S formed some granular structure in the cytoplasm (Figures 8A,B), which might relate to the intramolecular folding of duTRAF3-S. We suspected that the folding made an exposure of duTARF3-S coil 3 region and then enabled duTRAF3-S to form oligomers easier. It could also explain why increasing the expression of duTRAF3-S resulted in a great loss of duTRAF3 self-association (Figure 7B). Ultimately, the hetero-oligomerization of duTRAF3-duTRAF3-S impairs the interaction between duTRAF3 and duMAVS/duTRIF in a dose-dependent manner, and this interaction is dramatically reduced when duTRAF3-S increases (Figures 7C,D). Our results were compatible with the following sequence of events (Figure 9). duRIG-I/duMDA5 or duTLR3 recognized specific conserved pathogen-associated molecular patterns in duck cells and then recruited duMAVS or duTRIF, respectively (Figure S4 in Supplementary Material), which in turn recruited duTRAF3 to induce type I IFN response. The inability of duTRAF3-S to bind duMAVS or duTRIF and the hetero-oligomerization of duTRAF3-S with duTRAF3 prevented the duTRAF3 homotrimeric structural formation, which was required for efficient formation of duMAVS/duTRIF-duTRAF3 complex, so the hetero-oligomerization between duTRAF3-S and duTRAF3-S inhibited the formation of duMAVS/duTRIF-duTRAF3 complex (Figure 7) and ultimately attenuated the ability of duTRAF3 to activate the production of IFN-β. The MAVS-TRAF3 complex formation is crucial for IFN response to RNA viruses, and in the early phase of viral infection, TRAF3 acts as a linker in the assembly of the active MAVS-TRAF3-TBK1 signaling complex that also includes NEMO (52). In the late termination stage of infection, LUBAC mediates the linear ubiquitination of NEMO that facilitates its interaction with TRAF3 and disrupts the MAVS-TRAF3 complex (52). Besides, it is reported that TRIM29 could negatively regulate the type I IFN production by degrading NEMO (53). Our results show that duTRAF3-S is highly expressed in the late stage of poly(I:C) stimulation (Figures 2B,C); hence, it is also worth further study whether the negative regulatory effect of duTRAF3-S is related to duck NEMO.

Duck TRAF3 (duTRAF3) and duTRAF3-S mRNAs were ubiquitously distributed in all analyzed tissues (Figure 2A), which was in accordance with its multiple functions in the immune system. We found that duTRAF3 was most highly expressed in the brain, liver and digestive systems, suggesting that duTRAF3 might have innate immune functions for rapid control of pathogen infection in these organs. Human TRAF3 protein is expressed ubiquitously, and abundant TRAF3 expression was observed in digestive systems, liver, and brain (54), indicating a similarity in tissue expression profiles between duTRAF3 and human TRAF3. It was likely that the tissue expression pattern of this gene was conserved from duck to human in accordance with their functional conservation in these two species.

Multiple genome-encode viral recognition receptors have been identified. Interestingly, all of the major viral recognition pathways characterized thus far require TRAF3 to initiate type I IFN production (9). Type I IFNs are among the first line of host defenses against virus infections (55). IFN-β is usually induced within hours after viral infection. Once it is synthesized, it functions in both autocrine and paracrine fashions to prevent the replication and spread of virus. TRAF3 is required for IFN-β production in response to RNA virus, and TRAF3^−/−^ cells severely impaired the production of IFN-β in response to virus infection in human cells (10, 12, 56). In this study, in poly(I:C)-stimulated DEFs, the expression of duTRAF3 and duTRAf3-S protein were upregulated. Overexpression of duTRAF3 in DEFs inhibited AIV, DTMUV, and VSV replication (Figures 2D,E and 3E), which might relate to the ability of duTRAF3 to upregulate the production of IFN-β (Figure 3C, left). Conversely, overexpression of duTRAF3-S in DEFs blocked the production of IFN-β (Figure 3C, right), which might contribute to promote viral replication. These data demonstrated that duTRAF3 and duTRAF3-S involved in regulation of duck innate immune responses against virus infection. AIV exploits duck as a natural host and reservoir, and it would be interesting to further investigate whether duTRAF3-S contribute to the AIV evasion from duck innate immune responses.

In conclusion, our study indicated that duTRAF3 played an important role in duck immune response against virus infection and described a new mechanism by which truncated proteins negatively regulated the production of IFN-β *via* changing the formation of the homo-oligomerization of wild molecules.

## Ethics Statement

All experiments with H5N1/HM viruses were performed in Animal Biosafety Level 3 laboratory. This study was carried out in accordance with the recommendations of BSL-3, Huazhong Agricultural University (HZAU). The protocol was approved by the BSL-3 of HZAU. Animals experiments were approved by HZAU Animal Care and Use Committee.

## Author Contributions

XW, WQ, and HZ designed and wrote the paper. XW, WQ, SS, YL, KG, and MJ performed the experiments, analyzed the data, and interpreted the results. All authors reviewed, revised, and approved the final manuscript.

## Conflict of Interest Statement

The authors declare that the research was conducted in the absence of any commercial or financial relationships that could be construed as a potential conflict of interest.

## Footnotes

^1^http://www.ebi.ac.uk/Tools/msa/clustalo/.

^2^http://espript.ibcp.fr/ESPript/ESPript/.

^3^http://smart.embl-heidelberg.de/.
