# Supplementary material for: Negative Regulation of Interferon-β Production by Alternative Splicing of Tumor Necrosis Factor Receptor-Associated Factor 3 in Ducks
Source: Front Immunol. 2018 Mar 1;9:409. doi: 10.3389/fimmu.2018.00409 (PMC5863512; doi:10.3389/fimmu.2018.00409)
Supplement: Supplementary file 1 [file image_1.PDF]

Supplementary Material

Negative Regulation of IFN-β Production by Alternative Splicing of Tumor necrosis factor receptor-associated factor 3 in Ducks

Xiaoqin Wei, Wei Qian, Suolang Sizhu, Yongtao Li, Kelei Guo, Meilin Jin and Hongbo Zhou*

*** Correspondence:**
Hongbo Zhou
hbzhou@mail.hzau.edu.cn

Figure S1. Alignment of the protein sequences of duTRAF3 and duTRAF3-S. The conserved domains are indicated: a ring finger, two TRAF-type zinc finger motifs, a coiled-coil domain and a conserved TRAF-C domain.

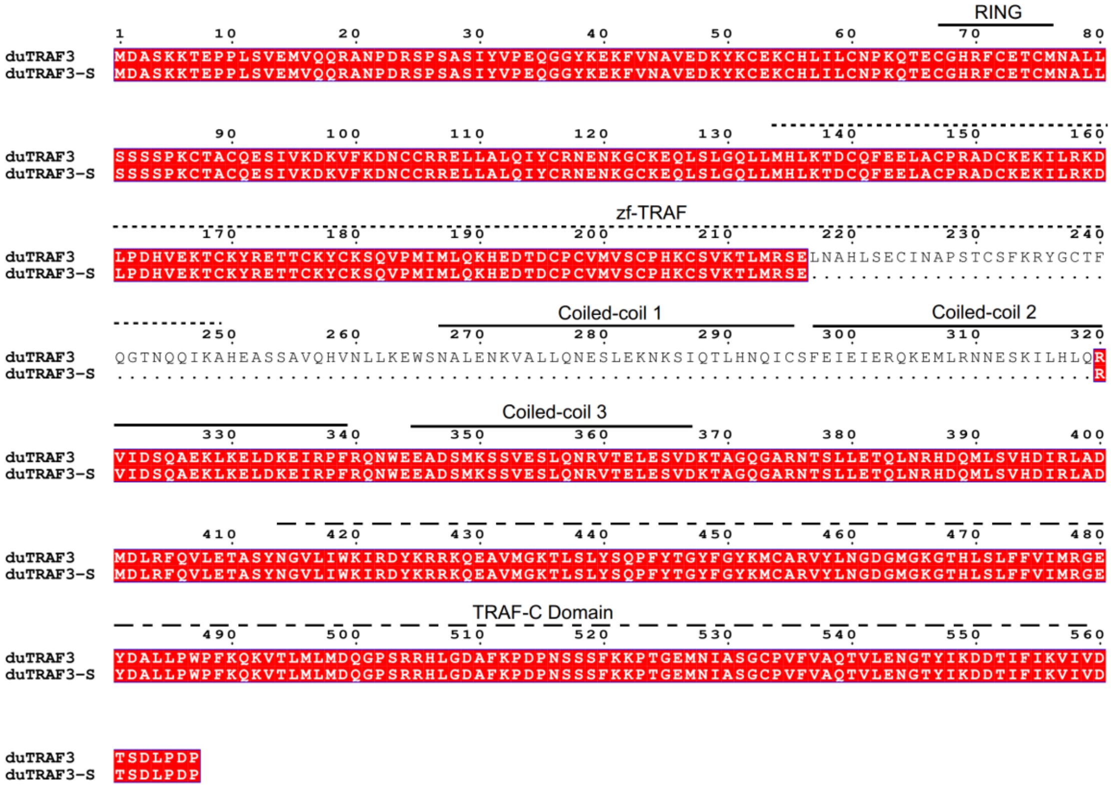

Figure S2. Multiple alignments of human, mouse, rat, porcine, duck, chicken, turkey, rock pigeon, zebra finch and zebra fish TRAF3 amino acid sequences. Alignment gaps were marked by dashes, different domain was marked. The GenBank accession numbers used in the comparison are human (NP_663777), mouse (NP_035762), rat

(NP_001102194), porcine (XP_005666500), duck (KX354824 and KX354825), chicken (XP_004936396), turkey (XP_003206786), rock pigeon (XP_005506142), zebra finch (XP_002200611) and zebra fish (NP_001003513).

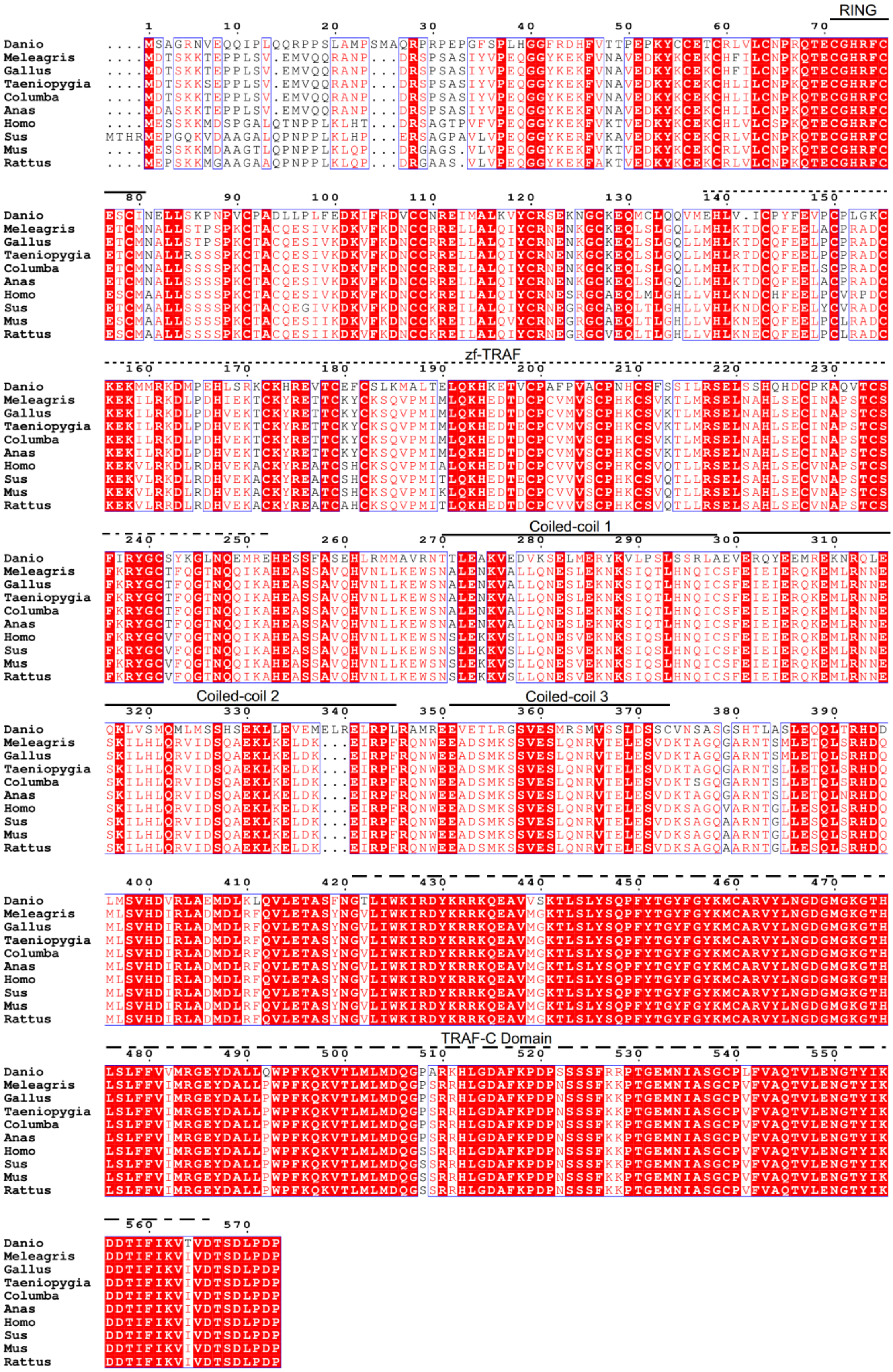

Figure S3. Phylogenetic analysis of avian, mammalian, and fish TRAF3 was carried out. The tree was constructed by the neighbor-joining (NJ) method using amino acid sequences aligned with MEGA 6. The scale bar indicated a branch length of 0.05.

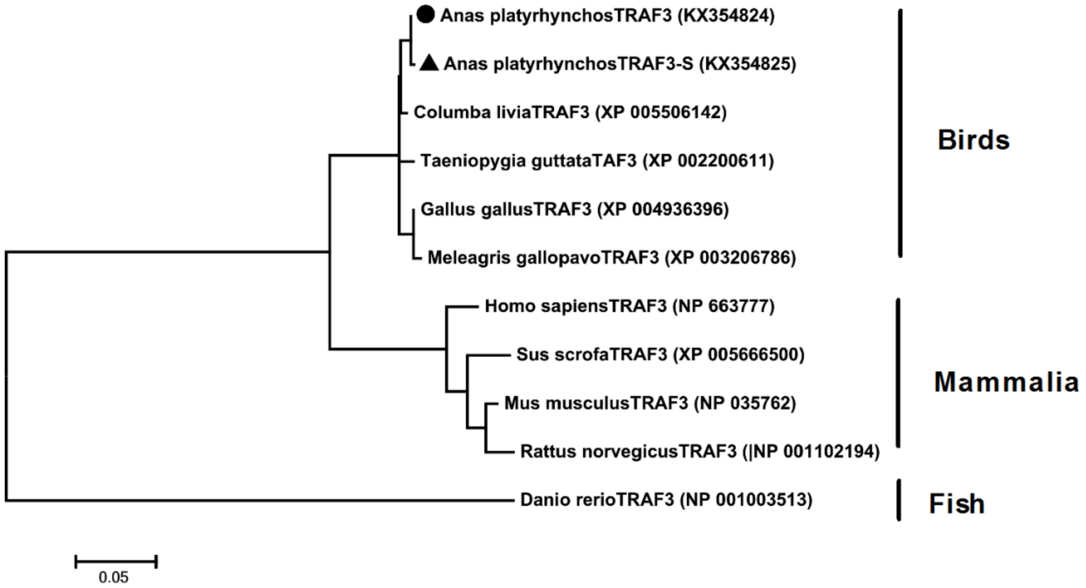

Figure S4. duMADA5 or duRIG-I interacts with duMAVS, duTLR3 interacts with duTRIF. 293T cells were cotransfected with Flag-duMAVS or Flag-duTRIF and the indicated expression plasmids. At 24 h posttransfection, cell extracts were collected and subjected to immunoprecipitation by anti-Flag. The cell extracts and the precipitated products were analyzed by immunoblotting with anti-Flag and anti-HA antibodies (Ab).

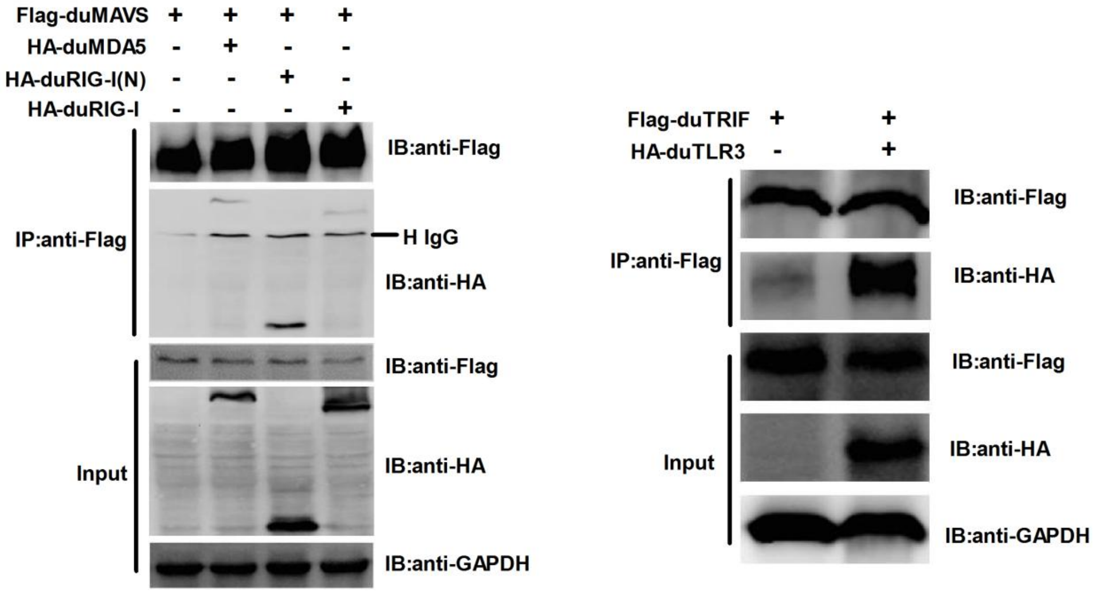

Table S1. Primers used in this study.

Primer name	Purpose		Sepuence of Oligonucleotied(5′-3′)	Accession No.
**pCAGGS-HA**				
duTRAF3	Full length	F	ggaattcATGGATGCCAGTAAGAAGACAG	XM_005020701.1
	Cloning	R	ccgctcgagTCAGGGGTCTGGTAGATCC	
T31-216	Truncated	F	ggaattcATGGATGCCAGTAAGAAGACAG	KX354824
	Cloning	R	ccgctcgagCTGTAAATGAAGTATTTTAGATTC	
T3217	Truncated	F	ggaattcTTGAATGCACATTTGTCAGA	
	Cloning	R	ccgctcgagTCAGGGGTCTGGTAGATCC	
T3319	Truncated	F	ggaattcCGAGTGATAGACAGTCAAGCA	
	Cloning	R	ccgctcgagTCAGGGGTCTGGTAGATCC	
**p3xFlag-CMV-14**				
duTRAF3	Full length	F	ggggtaccATGGATGCCAGTAAGAAGACAG	KX354824
	Cloning	R	ccgctcgagGGGGTCTGGTAGATCC	
RIG-I	Full length	F	ggggtaccATGACGGCGGACGAGAAGCGGAG	EU363349
	Cloning	R	ccgctcgagAAATGGTGGGTACAAGTTGG	
RIG-I(N)	Full length	F	ggggtaccATGACGGCGGACGAGAAGCGGAG	
	Cloning	R	ccgctcgagCTTCTTTGTTTCATAGACAGGTGGAG	
MDA5	Full length	F	ggggtaccATGTCGACGGAGTGCCGAGACG	KJ451070.1
	Cloning	R	ccgctcgagCTATCCTGTATTTCCACTTAAAT	
MAVS	Full length	F	ggaattcATGGGCTTCGCGGAGGAC	KJ466052
	Cloning	R	cgggatccTTTCTGCAGCCGGGCGT	
TRIF	Full length	F	cccaagcttATGAAGTACAACCAGAACATCAA	KX426571
	Cloning	R	cgggatccGGAGTTCTCCGTGGATTCTT	
duTRAF3	qRT-PCR	F	GCTGCACTTTTCAGGGAACA	KX354824
		R	TTCCGCAGCATTTCCTTCTG	
duTRAF3-S	qRT-PCR	F	ATGAGGAGCGAGCGAGTGATAGA	KX354825
		R	TGCCTGTTCAGCTGTGTCTC	
IFN-β	qRT-PCR	F	CACCTCCTCCAACACCTCTT	KM035791.1
		R	TGGAGGAAGTGTTGGATGCT	
NP (HM)	qRT-PCR	F	GCGTTCAGCCCACTTTCTCG	EU594350
		R	GGGTTCGTTGCCTTTTCGTC	
GAPDH	qRT-PCR	F	AAATTGTCAGCAATGCCTCTTG	XM_005016745.2
		R	TGGCATGGACAGTGGTCATAA	
**Split firefly luciferase complementation assay**				
N_{Luc}	two-step assembly PCR	F	ggaattcGAAGACGCCAAAAACATAAAGAAAGGCCCG	
		R	ACTTCCACCGCCTCCAGAACCTCCTCCACCTCCATCCTTGTCAAT	
duTRAF3		F	GGAGGCGGTGGAAGTGGTGGCGGAGGTAGCATGGATGCCAGTAAG	
		R	ACATGCTACCTCCGCCACCACTGGTACCGCCTCCAGAACCTCCTCC ACCGGG	
C_{Luc}		F	GGTACCAGTGGTGGCGGAGGTAGCATGTCCGGTTATGTA	
		R	ccgctcgagTCACACGGCGATCTTTCCGCCCTTCTTGGCCT	
